# Author Correction: Ontogenetically distinct neutrophils differ in function and transcriptional profile in zebrafish

**DOI:** 10.1038/s41467-023-41038-7

**Published:** 2023-08-31

**Authors:** Juan P. García-López, Alexandre Grimaldi, Zelin Chen, Claudio Meneses, Karina Bravo-Tello, Erica Bresciani, Alvaro Banderas, Shawn M. Burgess, Pedro P. Hernández, Carmen G. Feijoo

**Affiliations:** 1 Fish Immunology Laboratory, Faculty of Life Science, Andres Bello University, Santiago, Chile; 2 Stem Cells & Development Unit, Institut Pasteur, 75015 Paris, France; 3 UMR CNRS 3738, Institut Pasteur, Paris, France; 4 CAS Key Laboratory of Tropical Marine Bio-Resources and Ecology, South China Sea Institute of Oceanology, Chinese Academy of Sciences, Guangzhou, China; 5 Millennium Nucleus Development of Super Adaptable Plants (MN-SAP), Santiago, 8331150 Chile; 6 Millennium Institute Center for Genome Regulation (CRG), Santiago, 8331150 Chile; 7 Departamento de Fruticultura y Enología, Facultad de Agronomía e Ingeniería Forestal, Pontificia Universidad Católica de Chile, Santiago, 7820436 Chile; 8 Departamento de Genética Molecular y Microbiología, Facultad de Ciencias Biológicas, Pontificia Universidad Católica de Chile, Santiago, 8331150 Chile; 9 Translational and Functional Genomics Branch, National Human Genome Research Institute, Bethesda, MD USA; 10 Institut Curie, Université PSL, Sorbonne Université, CNRS UMR168, Laboratoire Physico Chimie Curie, 75005 Paris, France; 11 Institut Curie, PSL Research University, INSERM U934/CNRS UMR3215, Development and Homeostasis of Mucosal Tissues Lab, Paris, France

**Keywords:** Imaging the immune system, Neutrophils, Myelopoiesis, Chemotaxis, Transcriptomics

Correction to: *Nature Communications* 10.1038/s41467-023-40662-7, published online 15 August 2023

In this article, Shawn M. Burgess was incorrectly denoted as being one of the equally contributing authors. The original article has been corrected.

