# Supplementary material for: Ontogenetically distinct neutrophils differ in function and transcriptional profile in zebrafish
Source: Nat Commun. 2023 Aug 15;14:4942. doi: 10.1038/s41467-023-40662-7 (PMC10427629; doi:10.1038/s41467-023-40662-7)
Supplement: Supplementary file 1 — Supplementary Information [file 41467_2023_40662_MOESM1_ESM.pdf]

Supplementary Figures

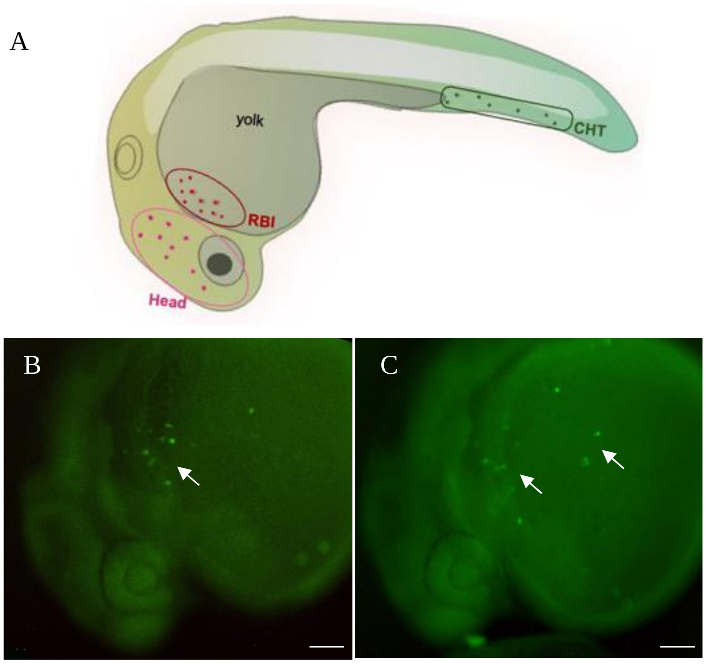

Supplementary Figure 1: (A) Scheme of a 24 hours post fertilization (hpf) embryo, showing the regions; rostral blood island (RBI); the head; or the caudal hematopoietic tissue (CHT); that were photoconverted. Dots in each region represent the resident neutrophils in each region. (B, C) Lateral view of the anterior region of a 20hpf Tg(*mpx*:Dendra2) embryo showing few and with low fluorescence intensity Dendra2^{+} cells (white arrow) at the RBI. Scale bar: 100 µm.

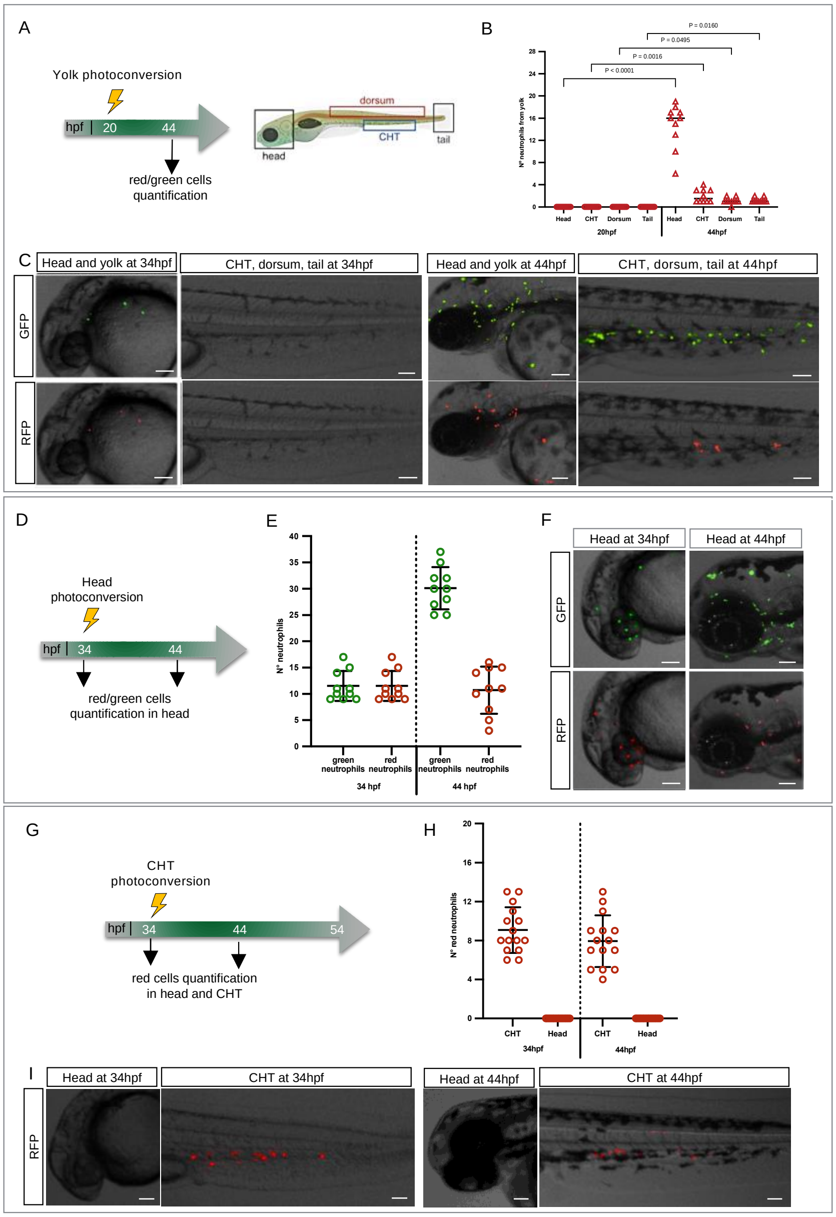

Supplementary Figure 2: Set up of RBI-derived and CHT-derived neutrophils differentiation strategy. (A) Experimental design. At 20 hours post fertilization (hpf), embryos with 0 neutrophils in the head and in the CHT were selected, and RBI-derived myeloid progenitors present at the yolk were photoconverted. Red and green neutrophils were quantified in the head, dorsum, CHT and tail at 44 hpf. (B) Quantification of photoconverted neutrophils present in different tissues in the larval body. n=10 embryos per experiment. (C) Representative images showing photoconverted (red cells) and non-photoconverted (green cells) neutrophils present in the CHT at 20 and 48 hpf. (D) Experimental design. At 34 hpf a photoconversion was performed in the head to label RBI-derived neutrophils red. (E) 0 and 20 hours post photoconversion (hpp), red and green neutrophils were quantified in the head. n=10 embryos per experiment. (F) Representative images showing photoconverted (red) and non-photoconverted (green) neutrophils in the head at 34 hpf and 44 hpf. (G) Experimental design. At 34 hpf neutrophils present in the CHT were photoconverted to label CHT-derived neutrophils red. (H) At 44 hpf red neutrophils present in the head and CHT were quantified. n=14 embryos per experiment. (I) Representative images showing red neutrophils only present at the CHT at 34 and 44 hpp. Statistical analysis was done with a two-sided Mann-Whitney U test. Experiments were performed independently at least 3 times. Scale bar: 100 µm.

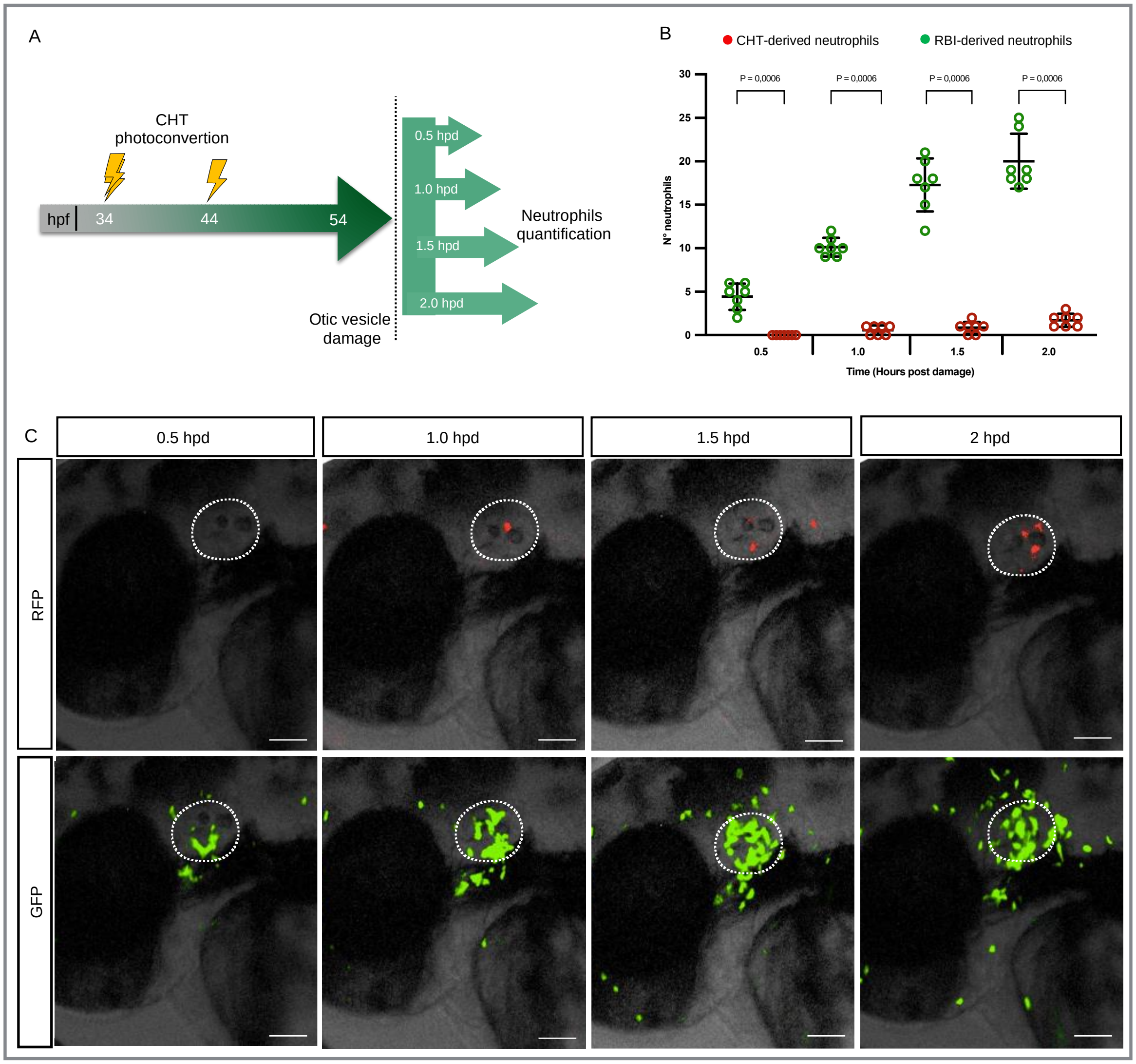

Supplementary Figure 3: Neutrophils recruitment to the otic vesicle after damage. (A) Experimental design. At 34 and 44 hpf, neutrophils located in the CHT were photoconverted and at 54 hpf, the otic vesicle was damaged. Green and red neutrophils present at the damaged zone were quantified at 0.5; 1; 1.5 and 2 hours post damage (hpd). (B) Quantification of RBI-derived and CHT-derived neutrophils present at the damaged area at the different time points analyzed. n=7 larvae per experiment. (C) Representative images showing CHT-derived photoconverted neutrophils (red cells) and RBI-derived non-photoconverted ones (green cells) recruited to the damaged area (purple circle) during the first 2 hpd. Statistical analysis was done with a two-sided Mann-Whitney U test. Data are represented as mean ± SD. Experiments were performed independently at least 3 times. Scale bar: 50 µm.

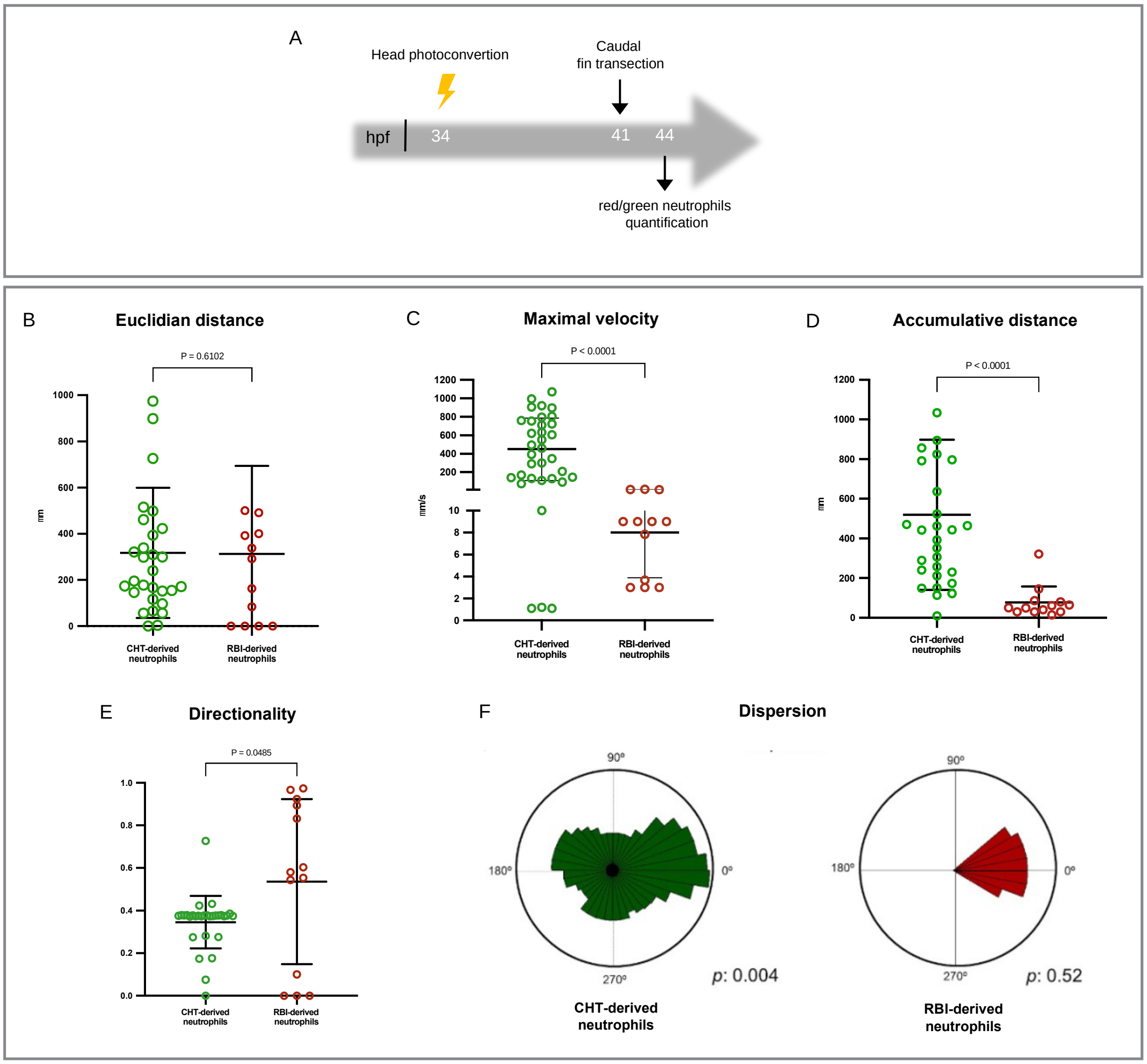

Supplementary Figure 4: Migration dynamic of RBI-derived and CHT-derived neutrophils during inflammation. (A) Experimental design. At 34 hpf the head was photoconverted to label RBI-derived neutrophils red. At 41 hpf, a caudal fin transection was made, and time lapse analysis was conducted for 3 hours post damage (hpd). Analysis of the (B) Euclidian distance; (C) maximal velocity; (D) cumulative distance and (E) directionally of RBI-derived and CHT-derived neutrophils recruited to the wound. n=10 larvae per experiment. Statistical analysis was done with a two-sided Mann-Whitney U test. Data are represented as mean ± SD. (F) Dispersion. Rose diagram showing the deviation angles in each segment of the route followed by neutrophils with respect to the direct route (0º) to the damage area. The p-values were obtained after applying a Rayleigh test to angles distribution in each subpopulation. n=10 larvae per experiment. Experiments were performed independently at least 3 times.

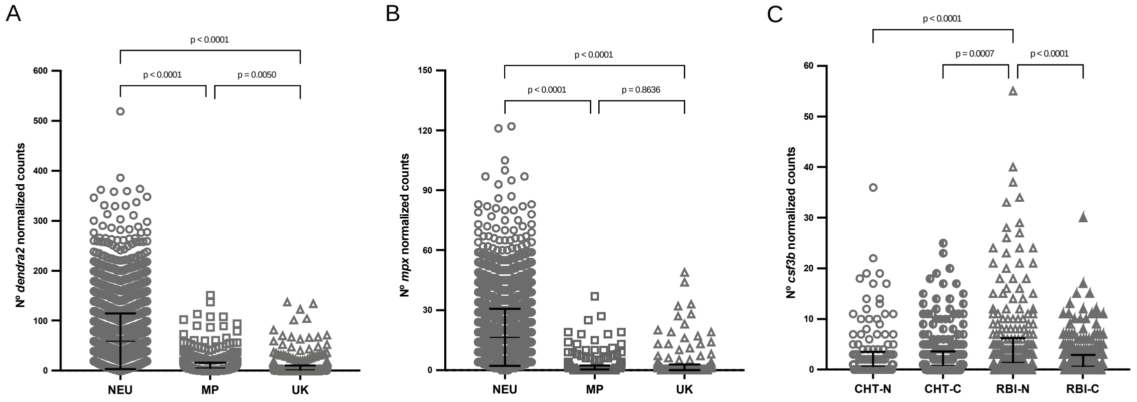

Supplementary Figure 5: Dendra2 is expressed mainly in neutrophils. Quantification of the number of *dendra2* (A) and *mpx* (B) normalized counts in neutrophils (NEU, N=3910), macrophage (MP, N=2028) and the unknow groups of cells (UK, N=1944). (C) *csf3b* normalized counts in CHT-derived normal (CHT-N, N=660), CHT-derived cut (CHT-C, N=853), RBI-derived normal (RBI-N, N=853), and RBI-derived cut (RBI-C, N=853). Statistical analysis was performed using ANOVA one-way and post-test Benjamini-Hochberg. Data are represented as mean ± SD.

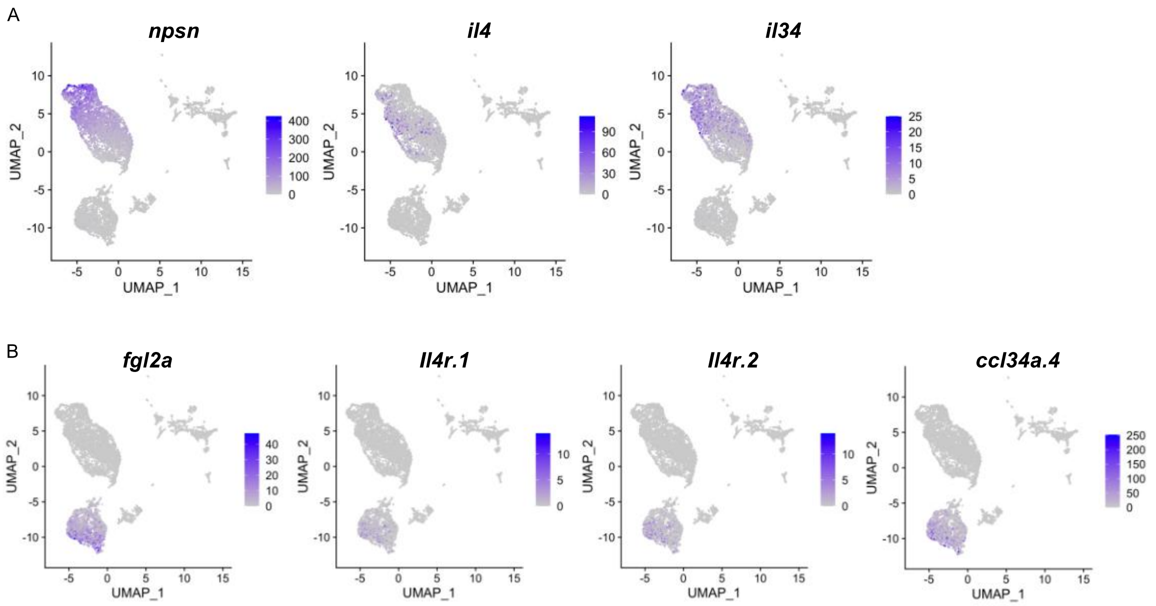

Supplementary Figure 6: Neutrophil and macrophage markers identified in our scRNAseq dataset. (A, B) UMAP plots showing expression of neutrophil-specific genes and macrophage-specific genes respectively.

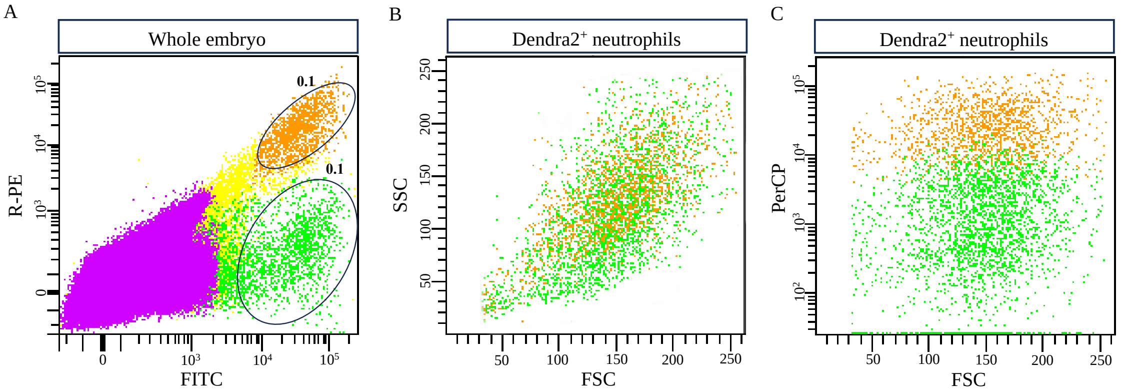

Supplementary Figure 7: Gating strategy to isolate Dendra2$^+$ neutrophils from whole embryo. Throughout the figure, Dendra2$^+$ non-photoconverted cells are labelled green and Dendra2$^+$ photoconverted cells are labelled orange. (A) Dendra2$^+$ cells (circled in black) were isolated based on fluorescence intensity. FITC were used for green fluorescence (non photoconverted cells) and R-PE for red fluorescence (photoconverted cells). Dendra2$^-$ events (violet) and Dendra2$^-$ SSC singlets (yellow) were discarded. Percentages are relative to total cells. (B) Dendra2$^+$ neutrophils based on light-scatter characteristics. (C) Dendra2$^+$ neutrophils based on fluorescence intensity and light-scatter characteristics. R-PE= R-Phycoerythrin; FITC= Fluorescein isothiocyanate; SSC= side scatter; FSC= forward scatter; PerCP= Peridinin Chlorophyll Protein Complex.
