# Supplementary material for: Ontogenetically distinct neutrophils differ in function and transcriptional profile in zebrafish
Source: Nat Commun. 2023 Aug 15;14:4942. doi: 10.1038/s41467-023-40662-7 (PMC10427629; doi:10.1038/s41467-023-40662-7)
Supplement: Supplementary file 3 — Description of Additional Supplementary Files [file 41467_2023_40662_MOESM3_ESM.pdf]

Description of Additional Supplementary Files

Supplementary Data 1. DE Lineages.

Supplementary Data 2. Results of the differential analysis of RBI-derived versus CHT-derived neutrophils steady state. Filtered on a minimum detection fraction threshold = 0.1, log fold-change threshold = 0.25 and p-value<0.05 (not adjusted), negative binomial test, out of the 2000 most variable genes, only genes with positive fold change were selected. (Columns; p_val: p-value, avg_log2FC: average log2 (fold change), pct.1: gene detection fraction in first group, pct.2: gene detection fraction in second group, p_val_adj: Bonferroni adjusted p-values, cluster: group in which this gene is upregulated)

Supplementary Data 3. Results of the differential analysis of RBI-derived neutrophils upon caudal fin transection. Filtered on a minimum detection fraction threshold = 0.1, log fold-change threshold = 0.25 and p-value<0.05 (not adjusted), negative binomial test, out of the 2000 most variable genes, only genes with positive fold change were selected. (Columns; p_val: p-value, avg_log2FC: average log2 (fold change), pct.1: gene detection fraction in first group, pct.2: gene detection fraction in second group, p_val_adj: Bonferroni adjusted p-values, cluster: group in which this gene is upregulated).

Supplementary Data 4. Results of the differential analysis of CHT-derived neutrophils upon caudal fin transection. Filtered on a minimum detection fraction threshold = 0.1, log fold-change threshold = 0.25 and p-value<0.05 (not adjusted), negative binomial test, out of the 2000 most variable genes, only genes with positive fold change were selected. (Columns; p_val: p-value, avg_log2FC: average log2 (fold change), pct.1: gene detection fraction in first group, pct.2: gene detection fraction in second group, p_val_adj: Bonferroni adjusted p-values, cluster: group in which this gene is upregulated)
