# Supplementary material for: Ontogenetically distinct neutrophils differ in function and transcriptional profile in zebrafish
Source: Nat Commun. 2023 Aug 15;14:4942. doi: 10.1038/s41467-023-40662-7 (PMC10427629; doi:10.1038/s41467-023-40662-7)
Supplement: Supplementary file 8 — Reporting Summary [file 41467_2023_40662_MOESM8_ESM.pdf]

nature portfolio

Corresponding author(s): Burgess, Hernandez, Feijoo

Last updated by author(s): 2023, 06, 22

Reporting Summary

Nature Portfolio wishes to improve the reproducibility of the work that we publish. This form provides structure for consistency and transparency in reporting. For further information on Nature Portfolio policies, see our Editorial Policies and the Editorial Policy Checklist.

Statistics

For all statistical analyses, confirm that the following items are present in the figure legend, table legend, main text, or Methods section.

n/a	Confirmed	
☐	☒	The exact sample size (*n*) for each experimental group/condition, given as a discrete number and unit of measurement
☐	☒	A statement on whether measurements were taken from distinct samples or whether the same sample was measured repeatedly
☐	☒	The statistical test(s) used AND whether they are one- or two-sided<br>*Only common tests should be described solely by name; describe more complex techniques in the Methods section.*
☒	☐	A description of all covariates tested
☐	☒	A description of any assumptions or corrections, such as tests of normality and adjustment for multiple comparisons
☐	☒	A full description of the statistical parameters including central tendency (e.g. means) or other basic estimates (e.g. regression coefficient) AND variation (e.g. standard deviation) or associated estimates of uncertainty (e.g. confidence intervals)
☒	☐	For null hypothesis testing, the test statistic (e.g. *F*, *t*, *r*) with confidence intervals, effect sizes, degrees of freedom and *P* value noted<br>*Give P values as exact values whenever suitable.*
☒	☐	For Bayesian analysis, information on the choice of priors and Markov chain Monte Carlo settings
☒	☐	For hierarchical and complex designs, identification of the appropriate level for tests and full reporting of outcomes
☒	☐	Estimates of effect sizes (e.g. Cohen's *d*, Pearson's *r*), indicating how they were calculated

Our web collection on statistics for biologists contains articles on many of the points above.

Software and code

Policy information about availability of computer code

Data collection	Matrices were generated using Cell Ranger 2.1.1 and mapping was performed using Ensembl gene annotation release 93. Specific functions and parameter values are detailed in the manuscript.
Data analysis	scRNAseq datasets were preprocessed using Seurat 3 in R. Doublets were inferred using DoubletFinder v3 (McGinnis et al., 2019). Gene set enrichment analyses were performed on those markers using Cytoscape and Cluego (Bindea et al., 2009). Specific functions and parameter values are detailed in the manuscript. Statistical analysis was performed using Prism 6.0 (GraphPad Software). Sample size was performed using G power 3.1

For manuscripts utilizing custom algorithms or software that are central to the research but not yet described in published literature, software must be made available to editors and reviewers. We strongly encourage code deposition in a community repository (e.g. GitHub). See the Nature Portfolio guidelines for submitting code & software for further information.

Data

Policy information about availability of data

All manuscripts must include a data availability statement. This statement should provide the following information, where applicable:

- Accession codes, unique identifiers, or web links for publicly available datasets
- A description of any restrictions on data availability
- For clinical datasets or third party data, please ensure that the statement adheres to our policy

The scRNAseq data generated in this study have been deposited in the Dryad database https://datadryad.org/stash/dataset/doi:10.5061/dryad.4mw6m90dj.

Human research participants

Policy information about studies involving human research participants and Sex and Gender in Research.

Reporting on sex and gender	not applicable
Population characteristics	not applicable
Recruitment	not applicable
Ethics oversight	not applicable

Note that full information on the approval of the study protocol must also be provided in the manuscript.

Field-specific reporting

Please select the one below that is the best fit for your research. If you are not sure, read the appropriate sections before making your selection.

☒ Life sciences ☐ Behavioural & social sciences ☐ Ecological, evolutionary & environmental sciences

For a reference copy of the document with all sections, see nature.com/documents/nr-reporting-summary-flat.pdf

Life sciences study design

All studies must disclose on these points even when the disclosure is negative.

Sample size	Sample size was determined performing a power analysis using software G Power 3.1 (Faul, Erdfelder, Lang and Buchner, 2007) according to Charan and Kantharia., 2013.
Data exclusions	no data was excluded
Replication	For all experiments, except for the single cell transcriptomic assay (scRNAseq), three biological replicates were performed. All attempts at replication were successful. The scRNAseq was performed one time since our unit of analysis is the cell (RBI-derived neutrophils v/s CHT-derived neutrophils), and in the assay performed we had hundreds of cells that allow us to have a high degree of confidence in the result obtained. Importantly, because scRNAseq requires destructive sampling of cells, standard experimental design approaches for disentangling technical and biological sources of variability are not applicable.
Randomization	Embryos or larvae were allocated randomly into control and experimental groups
Blinding	Investigators were blinded to group allocation during data collection and analysis

Reporting for specific materials, systems and methods

We require information from authors about some types of materials, experimental systems and methods used in many studies. Here, indicate whether each material, system or method listed is relevant to your study. If you are not sure if a list item applies to your research, read the appropriate section before selecting a response.

Materials & experimental systems

n/a	Involved in the study	
☒	☐	Antibodies
☒	☐	Eukaryotic cell lines
☒	☐	Palaeontology and archaeology
☐	☒	Animals and other organisms
☒	☐	Clinical data
☒	☐	Dual use research of concern

Methods

n/a	Involved in the study	
☒	☐	ChIP-seq
☒	☐	Flow cytometry
☒	☐	MRI-based neuroimaging

Animals and other research organisms

Policy information about studies involving animals; ARRIVE guidelines recommended for reporting animal research, and Sex and Gender in Research

Laboratory animals	2, 3, 4 and 5 days post fertilization Tg(mpx:Dendra2) transgenic zebrafish (Danio rerio) embryos/larvae were used
Wild animals	no wild type animals were used.
Reporting on sex	not applicabe. At the used stage of development the gender is not identifiable
Field-collected samples	no field-collected samples were used
Ethics oversight	Ethics Committee of the Universidad Andres Bello.

Note that full information on the approval of the study protocol must also be provided in the manuscript.
